# Complete Genome Sequence of Pseudomonas coronafaciens pv. oryzae 1_6

**DOI:** 10.1128/MRA.01564-19

**Published:** 2020-01-16

**Authors:** David A. Baltrus, Meara Clark

**Affiliations:** a School of Plant Sciences, University of Arizona, Tucson, Arizona, USA; b School of Animal and Comparative Biomedical Sciences, University of Arizona, Tucson, Arizona, USA; Georgia Institute of Technology

## Abstract

Pseudomonas coronafaciens pv. oryzae 1_6 was originally isolated as a phytopathogen of rice. Here, we report a complete genome sequence for this strain, containing a circular chromosome and one circular plasmid, assembled using a hybrid approach combining Illumina paired-end reads and longer reads sequenced on an Oxford Nanopore Flongle flow cell.

## ANNOUNCEMENT

Strains classified in the Pseudomonas syringae *sensu lato* complex are important pathogens of many agriculturally important crops (1). Phylogenetic comparisons of P. syringae *sensu lato* have split this complex into at least 13 separate phylogroups (2, 3). Pseudomonas coronafaciens pv. oryzae strain 1_6 (formerly referred to as P. syringae pv. oryzae strain 1_6) was the first sequenced member of phylogroup 4 and was originally isolated as a phytopathogen of rice in Japan (4). Ten years after the first report of a draft genome sequence for this strain, we report the complete genome sequence of P. coronafaciens pv. oryzae 1_6. This genome was assembled using an independent approach (4) of blending Illumina paired-end reads with those generated on an Oxford Nanopore Flongle flow cell.

The lyophilized isolate of strain P. syringae pv. oryzae 1_6 was originally acquired from the Ministry of Agriculture, Fisheries, and Forestry in Japan (MAFF number 311107) by the Baltrus lab, resuspended in King’s B (KB) medium upon receipt, and streaked to single colonies on KB agar plates. A single colony arising from this original plating then was picked to 2 ml KB broth and grown overnight at 27°C, at which point an aliquot was frozen at –80°C in 40% glycerol to create a stock culture. This original frozen stock was then again streaked as single colonies onto KB agar plates and picked to KB broth to generate a second stock culture prepared as described above. For each genomic DNA extraction used in the assembly reported here, a sample of this second frozen stock was streaked onto KB agar plates, and a single colony was transferred to 2 ml of KB broth and grown overnight at 27°C in a shaking incubator at 220 rpm. Genomic DNA used for Illumina sequencing was isolated from a 2-ml overnight culture via the Promega (Madison, WI) Wizard kit following the manufacturer’s protocols. Genomic DNA used for Nanopore sequencing was isolated from a separate 2-ml overnight culture via the Promega Wizard kit, followed by selection for longer fragments using the Circulomics (Baltimore, MD) short-read extractor kit following the manufacturer’s protocols. RNase was added per the manufacturer’s protocols for each of the genomic isolations.

Genomic DNA was sequenced by MiGS (Pittsburgh, PA) following the standard workflow for library preparation and read trimming. As described in reference 5, this workflow uses an Illumina tagmentation kit for library generation, followed by sequencing on a NextSeq 550 instrument with 150-bp paired-end reads. Trimmomatic (6) was used for adaptor trimming using the default settings. This workflow generated a total of 1,389,168 paired reads and 357,148,894 bp (∼60× coverage) of sequence.

A separate isolation of genomic DNA was sequenced by the Baltrus lab with an Oxford Nanopore MinION system using a Flongle flow cell, with 500 ng of DNA prepared using the LSK-109 kit without shearing. Reads were called during sequencing using Guppy version 3.2.6 using a MinIT device (ont-minit-release 19.10.3) for processing. Sequencing on the MinION system using a Flongle flow cell (FLG001, flow cell number ABE077) generated 61,803 reads for a total of 363,833,842 bp (∼60× coverage) of sequence with a read *N*_50_ value of 11,425 bp.

Hybrid assembly of both read types was performed using Unicycler version 0.4.8 (7) and resulted in a single chromosome (5,662,937 bp) and a plasmid, pPor1_6 (51,521 bp), with a 57.9% GC content. Both replicons were circular as called by Unicycler. These chromosome and plasmid sequences were annotated using the NCBI PGAP (8) and are predicted to together contain 5,220 genes representing 4,976 protein-coding sequences, 2 complete sets of rRNAs (5S, 16S, and 23S rRNAs) and 1 additional 5S rRNA gene copy, 63 tRNAs, and 4 noncoding RNAs (ncRNAs). Default parameters were used for all software.

### Data availability.

This genome project is indexed at GenBank under BioProject accession number PRJNA31357. The chromosomal sequence for P. coronafaciens pv. oryzae 1_6 can be found at GenBank under the accession number CP046035, while the sequence for plasmid pPor1_6 can be found under the accession number CP046036. The Illumina reads can be found at SRA accession number SRX7191131. Fast5 files from the MinION Flongle run can be found at SRA accession number SRX7191132. A log file generated with Unicycler for an assembly of this genome as well as a graph file (.gfa) resulting from this assembly can be found at Figshare (https://doi.org/10.6084/m9.figshare.6025748).
